# Off-Resonance Gold Nanobone Films at Liquid Interface for SERS Applications

**DOI:** 10.3390/s22010236

**Published:** 2021-12-29

**Authors:** Rebeca Moldovan, Valentin Toma, Bogdan-Cezar Iacob, Rareș Ionuț Știufiuc, Ede Bodoki

**Affiliations:** 1 Analytical Chemistry Department, Faculty of Pharmacy, Iuliu Hațieganu University of Medicine and Pharmacy, 400349 Cluj-Napoca, Romania; rebeca.magda@umfcluj.ro (R.M.); iacob.cezar@umfcluj.ro (B.-C.I.); 2 MedFuture Research Center for Advanced Medicine, Iuliu Hațieganu University of Medicine and Pharmacy, 400349 Cluj-Napoca, Romania; valentin.toma@umfcluj.ro (V.T.); rares.stiufiuc@umfcluj.ro (R.I.Ș.); 3 Pharmaceutical-Biophysics Department, Faculty of Pharmacy, Iuliu Hațieganu University of Medicine and Pharmacy, 400349 Cluj-Napoca, Romania

**Keywords:** SERS, nanobones, nanorods, self-assembly, resonance, liquid interface

## Abstract

Extensive effort and research are currently channeled towards the implementation of SERS (Surface Enhanced Raman Spectroscopy) as a standard analytical tool as it has undisputedly demonstrated a great potential for trace detection of various analytes. Novel and improved substrates are continuously reported in this regard. It is generally believed that plasmonic nanostructures with plasmon resonances close to the excitation wavelength (on-resonance) generate stronger SERS enhancements, but this finding is still under debate. In the current paper, we compared off-resonance gold nanobones (GNBs) with on-resonance GNBs and gold nanorods (GNRs) in both colloidal dispersion and as close-packed films self-assembled at liquid-liquid interface. Rhodamine 6G (R6G) was used as a Raman reporter in order to evaluate SERS performances. A 17-, 18-, and 55-fold increase in the Raman signal was observed for nanostructures (off-resonance GNBs, on-resonance GNBs, and on-resonance GNRs, respectively) assembled at liquid-liquid interface compared to the same nanostructures in colloidal dispersion. SERS performances of off-resonance GNBs were superior to on-resonance nanostructures in both cases. Furthermore, when off-resonance GNBs were assembled at the liquid interface, a relative standard deviation of 4.56% of the recorded signal intensity and a limit of detection (LOD) of 5 × 10^−9^ M could be obtained for R6G, rendering this substrate suitable for analytical applications.

## 1. Introduction

SERS represents a valuable analytical tool for the fingerprint-type, trace detection of molecules located in close proximity of a plasmonic substrate. To this day, it is still debated whether off-resonance or on-resonance nanostructures generate stronger SERS enhancement [1,2]. Many types of SERS substrates (metallic colloids in solution, substrates built by nano-lithography or self-organization) have been described for a diversity of applications in various fields of life sciences, with substantial efforts being directed towards the development of diagnostic tools [3,4,5,6]. Nevertheless, an ongoing pursuit of new plain or composite metal structures of diverse morphologies and/or self-assembled or aggregated nanostructures is observed, which could further serve as analytical platforms with even more improved sensitivity. Amongst these, anisotropic metal nanoparticles, such as rods, stars, cubes, prisms, and nanoplates are ideal candidates due to the high electromagnetic field enhancements at their sharp edges, known as the “lighting rod effect” or “plasmonic antenna effect” [7].

Among nanostructured noble metal colloids, gold nanorods (GNRs) are considered attractive and highly versatile candidates for SERS applications. This is attributable to their good chemical stability and valued plasmonic properties, which can be easily tuned throughout the visible to near-infrared range by varying the local dielectric environment (surrounding medium and/or surface modification), their size, aspect ratio, or aggregation state [8]. Being anisotropic nanostructures, the oscillation of the conduction electrons on the short and long axis of the rod generates two surface plasmon resonance absorption bands. The transversal one is situated at approximately 520 nm and the longitudinal one, characteristic to GNRs, is related to their aspect ratio (length/width ratio). Although GNRs are intensely investigated for a wide range of sensing applications [9,10,11,12], their utility as colloidal SERS substrates is somewhat limited, as Raman signal amplification is conditioned by a perpendicular orientation of the laser to the long axis of the rod [13]. Consequently, in colloidal dispersion, due to the random orientation of the rods, the overall signal enhancement efficiency is far from ideal. These findings are also in accordance with near field discrete dipole approximation (DDA) simulations of bare GNRs [14].

As of late, a new class of nanostructures derived from GNRs with improved geometry was developed for SERS detection, namely GNBs. Although GNBs seem to be still underexplored among gold nanostructures, by virtue of their particular shape, the Raman signal amplification occurs regardless of the laser’s direction. In contrast to conventional rods, hot-spots are generated in the gap region between the lobes when the laser is parallel to their long axis [13]. Numerous methods for their synthesis have been reported, such as the overgrowth of GNRs by excess of reducing agent [15], the aging of GNRs [16], pH adjustment [17,18], or the adjustment of silver/iodide ratio [19]. As the growth mechanism of GNRs or GNBs is still not completely understood [20], their lab-scale synthesis cannot be fully standardized, requiring slight adaptations of the methodology by each user.

Additional Raman signal enhancement, for superior detection sensitivity, may be achieved by assembling or aggregating nanostructures. When two GNRs are in proximity, the electromagnetic field surrounding them is highly amplified. This condition is relatively easy to reach at a liquid-liquid interface. In order to reduce the superficial tension, nanostructures may be directed at the liquid interface by electrostatic interactions [21,22] (assembly) or reduction of the solvent’s dielectric constant (aggregation). Wang et al. described for the first time the aggregation of GNRs in a thin film at the water/toluene interface [23]. When ethanol [24,25] is used as an inducer, the nanostructures aggregate in close packed arrays and show strong plasmonic coupling resulting in a multitude of SERS “hot spots” over the detection zone. Nanostructures are destabilized upon the addition of ethanol due to a decrease of surface charge density. Consequently, they are directed and trapped at the interface in order to diminish the interfacial energy between the two liquids. These self-assembled or aggregated films can be used in situ [26] or can be further transferred onto solid substrates for SERS analysis. Building nanoplatforms at a liquid interface is advantageous due to a larger stability compared to suspended aggregates, offering a time-effective alternative for the detection of various analytes [22,27,28], without the necessity of transferring the film onto a solid substrate.

The aim of the current study was to evaluate the SERS signal enhancement efficiency of off-resonance GNBs compared with on-resonance GNBs and GNRs, both in colloidal dispersion and as close-packed films generated at liquid-liquid interface. Moreover, we monitored the time-dependent morphological evolution of GNBs upon synthesis and the associated changes in their plasmonic properties in order to estimate the acceptable time window for their analytical usability.

## 2. Materials and Methods

### 2.1. Chemicals

Cetyltrimethylammonium bromide (CTAB, >98%), silver nitrate (AgNO_3_, >99%), L-ascorbic acid (AA, >99%), chloroauric acid tetrahydrate (HAuCl_4_·4H_2_O), *n*-hexane (≥95%), diethyl ether (≥98.0%), trichloro (1H,1H,2H,2H-perfluorooctyl) silane (97%), rhodamine 6G (99%), ethyl alcohol (≥99.9%), and conventional gold nanorods (aqueous dispersion, CTAB stabilizer < 0.1%) were purchased from Sigma-Aldrich (Steinheim, Germany). Sodium borohydride (NaBH_4_, 99%), sulfuric acid (H_2_SO_4_ 98%), and hydrogen peroxide (H_2_O_2_ 30%) were purchased from Merck (Darmstadt, Germany). All chemicals were used as received with no further purification. Ultrapure water (18 MΩ cm^−1^, EasyPure RoDi, Barnstead, UK) was used for the preparation of all aqueous solutions.

### 2.2. Synthesis of Gold Nanobones

GNBs were synthesized by an adapted seed-mediated growth method [29].

Gold seeds were synthetized as follows: 60 µL of a 0.01 M aqueous solution of HAuCl_4_·3H_2_O was added to 1.8 mL, 0.1 M solution of CTAB in a plastic tube. The mixture was vortexed for one minute. Next, 300 µL of freshly prepared ice-cold 0.01 M NaBH_4_ solution was added all at once, followed by vigorous stirring for one minute. The color of the solution changed from bright yellow to a dark brown-yellow color instantly. The test tube was left undisturbed for two hours at a constant temperature of 27 °C. Before further use, the gold seeds were diluted 10 times.

Off-resonance GNBs with the longitudinal plasmon band at 710 nm (sample A) were synthetized as follows: 200 µL HAuCl_4_ 0.01 M was added to 4.75 mL CTAB 0.1 M in a plastic tube, followed by one-minute stirring. Then, 37.5 µL AgNO_3_ 0.01 M was added to the solution, followed by the addition of 40 µL AA 0.1 M and mixing. At this moment, the solution became colorless. Finally, 25 µL of seed solution was added and the mixture was stirred for one minute. The test tube was left undisturbed at a temperature of 27 °C for 24 h in order to allow the growth process to complete.

On-resonance GNBs with the longitudinal plasmon band at 765 nm (sample B) were synthesized in the exact same manner, with the note that 150 µL of the seed solution were added in the growth solution.

### 2.3. Stability Assessment

To evaluate the stability of GNBs, a synthesized batch was sedimented by centrifugation (30 min at 7800 rpm) and redispersed in ultrapure water. The colloid was then maintained in a dry-heat oven at 25 °C and analyzed periodically by UV-Vis spectroscopy and TEM imaging up to 14 days. These experiments were performed in triplicate. 

### 2.4. Transmission Electron Microscopy

To perform electron microscopy, HT7700 (Hitachi, Tokyo, Japan) Transmission Electron Microscope (TEM) was employed, operating at 100 kV (using high-resolution operation mode). On a 400-mesh carbon-coated copper grid, 8 µL of each sample was spotted and left undisturbed for five minutes. The excess solution was then blotted away with ashless filter paper, and the grids were left enclosed at room temperature in order to completely dry.

### 2.5. SERS Measurements in Dispersion and at Liquid Interface

A Renishaw™ inVia Reflex Raman (Renishaw plc, Gloucestershire, UK) confocal multilaser spectrometer was used to measure Raman and SERS spectra with a resolution of ~1 cm^−1^. A 100× (N.A = 0.85) objective lens was used to record the Raman spectra and 5× (N.A = 0.12) for SERS measurements. A 785 nm diode laser was used for excitation. The spectrograph was equipped with a 1200 lines/mm grating and a charge coupled device camera (CCD). Baseline correction was performed with the Wire 4.2 software provided by Renishaw and it was applied to all Raman/SERS spectra to eliminate the fluorescence background. Each spectrum represents the average of minimum five spectral acquisitions, collected on different points of the sample.

SERS measurements were performed on the synthesized GNBs (off-resonance—sample A and on-resonance—sample B) and compared with those recorded on commercial GNRs having the longitudinal plasmon band located at 765 nm (on-resonance—sample C), in both colloidal dispersion and close packed films at liquid interface. All synthetized GNBs used as SERS substrates were washed once by centrifugation at 7800 rpm for 30 min and redispersed in water. All samples were then concentrated by centrifugation and brought to the same optical density.

Rhodamine 6G (R6G) was used as Raman reporter to compare the enhancement effects of the studied nanostructures. In the case of colloidal dispersions, 5 µL of GNBs/GNRs was mixed with 5 µL of R6G 0.1 mM. Next, 5 µL of the resulting mixture were placed on aluminum foil attached to a microscope glass slide. The laser power (measured at sample surface) was 71.6 mW, and the exposure time was set to 10 s. To sample the aggregated nanostructured layer at a liquid interface, 50 µL of GNBs/GNRs was mixed with 50 µL R6G 0.1 mM in a hydrophobized glass vial, followed by the addition of 150 µL hexane. Subsequently, upon the addition of 300 µL of ethanol, a metallic film developed instantly at the interfacial region of the aqueous and organic phase (Figure S2). The SERS signal was collected at the liquid-air interface upon the evaporation of hexane. The laser power (measured at the sample surface) was 2.48 mW, and the exposure time was set to 10 s.

The surface modification of glass vials was performed according to the method described by Ma Yongmei et al. [27]. Briefly, the glass surface was first thoroughly cleaned by immersion in piranha solution (H_2_SO_4_ 98%:H_2_O_2_ 30% = 3:1 (*v*/*v*)) for 1 h, followed by an extensive rinsing with ultrapure water. Finally, the vials were dried in a stream of nitrogen and further placed in a dry-heat oven for 1 h at 110 °C. For surface hydrophobization the vials were immersed in 40 mM trichloro (1H,1H,2H,2H-perfluorooctyl) silane in diethyl ether for 12 h, followed by a successive washing with ethanol and water and a final drying in the oven (1 h, 110 °C).

## 3. Results and Discussion

### 3.1. Morphology and Optical Properties of Gold Nanorods and Nanobones

UV-Vis spectra revealed a common resonance plasmonic band at approximately 520 nm for both GNBs (samples A and B) and GNRs (sample C). This band is almost independent of the aspect ratio of these nanostructures and corresponds to the transversal oscillatory mode of the electrons. In contrast, the longitudinal plasmon band is strongly dependent on the aspect ratio (Table 1). Even though the studied nanostructures (samples B and C) have different sizes and morphologies (GNBs and GNRs respectively), considering as width the middle of the rod, they share the same aspect ratio and, consequently, the same longitudinal plasmon resonance wavelength.

**Figure 1:**
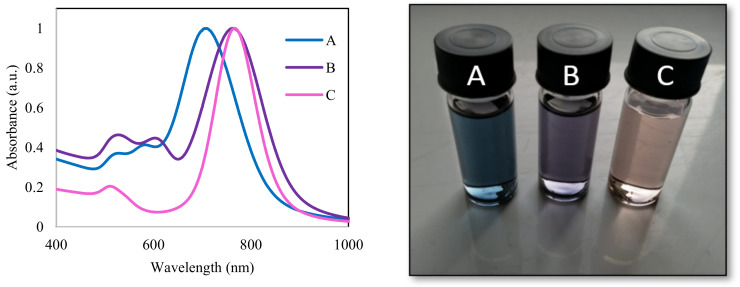
Normalized absorption spectra and corresponding picture of the studied anisotropic gold nanostructures (**A**—off-resonance GNBs, **B**—on-resonance GNBs, **C**—on-resonance GNRs).

Interestingly, in the case of GNBs (samples A and B), a third resonance plasmonic band can be observed in the UV-Vis spectra (Figure 1), in-between the transversal and longitudinal ones, and corresponds to an additional resonant oscillatory mode of electrons. These results are consistent with simulations and the work of other authors [13,16,30].

**Figure 2:**
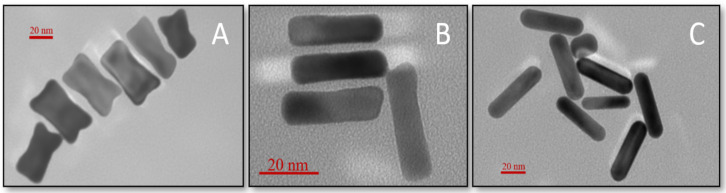
TEM images of synthetized GNBs (**A** and **B**) and commercial GNRs (sample **C**).

**Figure 3:**
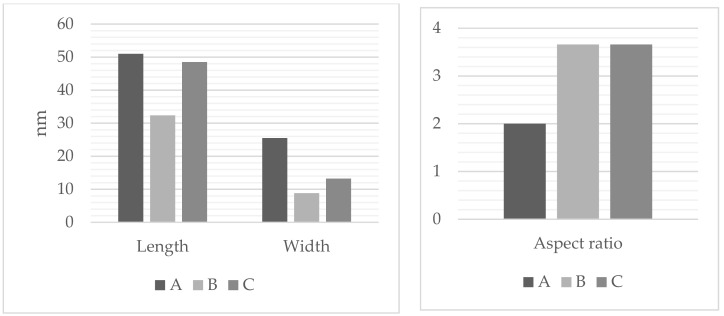
Dimensions of the studied anisotropic gold nanostructures.

TEM analysis (Figure 2) confirmed the spectroscopic data and the proposed morphology of the studied nanostructures. Dimensions and optical properties of samples A, B, and C are summarized in Figure 3 and Table 1, respectively.

### 3.2. Stability of Gold Nanobones

Studies show that, under optimal conditions, GNRs in aqueous dispersions are stable for at least nine months [31]. Nevertheless, to the best of our knowledge, comprehensive studies on GNBs are lacking and their stability in time has not been reported. It is expected that owing to the emphasized negative curvatures found at the tips of the rods, they evolve to shapes that are more energetically favorable [32].

Consequently, the morphological modifications and the changes in the corresponding optical properties of synthetized GNBs during storage at room temperature (25 °C) were monitored for 14 days.

**Figure 4:**
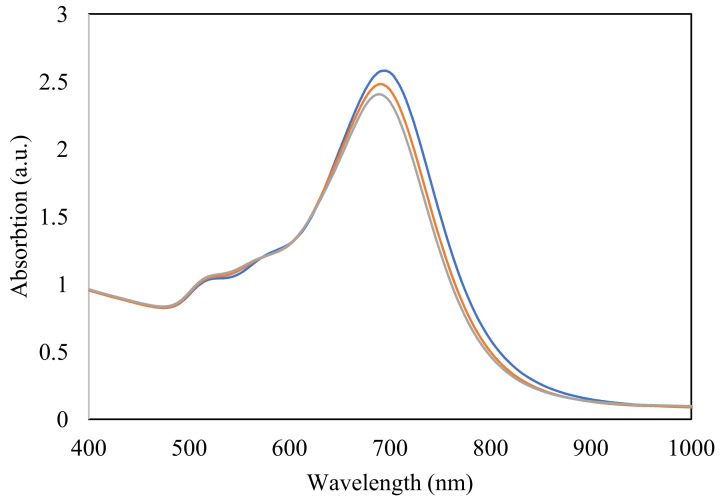
Absorbance spectra of GNBs right after synthesis (blue), and after 7 (orange), and 14 (grey) days of storage at room temperature.

A slight decrease in the absorbance spectra of GNBs was recorded after 7 days of storage (Figure 4) most likely due to minor aggregation effects. The unchanged allure of the absorption spectra and the lack of spectral shifts of the third resonance plasmonic band suggest no morphological changes of GNBs within this timeframe. However, after another 7 days, a further decrease in the absorbance of the main plasmonic band and the concurrent fade-out of the third plasmonic band characteristic to GNBs was observed (Figure 4), suggesting morphological alterations.

**Figure 5:**
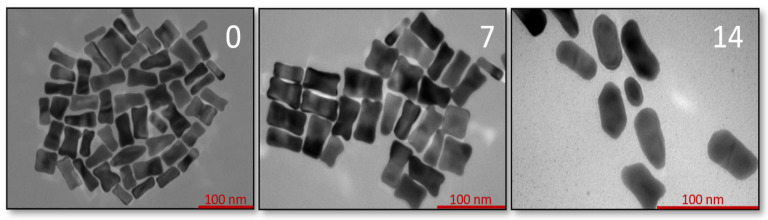
TEM images of GNBs—after synthesis (0), 7, and 14 days of storage at room temperature.

**Figure 6:**
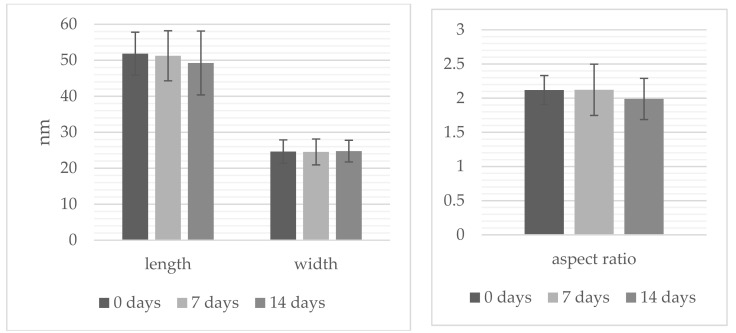
Time dependent morphological changes of studied nanostructures.

TEM results (Figure 5) were consistent with the changes observed in the absorbance spectra, morphological alterations being evident after 14 days, with an obvious smoothing of the edges. The latter phenomenon may be considered as the natural time evolution of nanostructures towards more stable geometries (Figure 6), doubled by the effect of residual reagents from the fabrication process [33]. These unreacted reducing agents alter the morphology, especially at the edges of the rods, where the stabilizing double layer of CTAB is less densely packed compared to other regions [34].

Neither the absorption spectra (no hyperchromic effect in the range of 800–1000 nm), nor the transmission electron micrographs revealed important signs of GNBs aggregation throughout the studied timeframe.

The morphological and optical integrity of GNBs were considered to be maintained for one week upon storage at 25 °C and thus, this time interval was considered for further experiments.

### 3.3. SERS Performances of Off-Resonance GNBs and On-Resonance GNBs and GNRs

Rhodamine 6G was used to compare the SERS performances of the studied nanostructures. Some of the characteristic vibrational bands of R6G (Figure S1) were identified at 612 cm^−1^ (C–C–C ring in-plane vibration), 774 cm^−1^ (C–H out of-plane bending), 1185 cm^−1^ (C–H in-plane bending), 1311 cm^−1^ (N–H in-plane bending), 1362 cm^−1^ and 1507 cm^−1^ (C–C stretching), respectively, corresponding to the literature [35]. As representative Raman bands, the C–C–C ring in-plane vibration mode located at 612 cm^−1^ was considered for the comparison of SERS activities of the studied gold nanostructures recorded in two different approaches: dispersion and aggregated films at the liquid-air interface.

**Figure 7:**
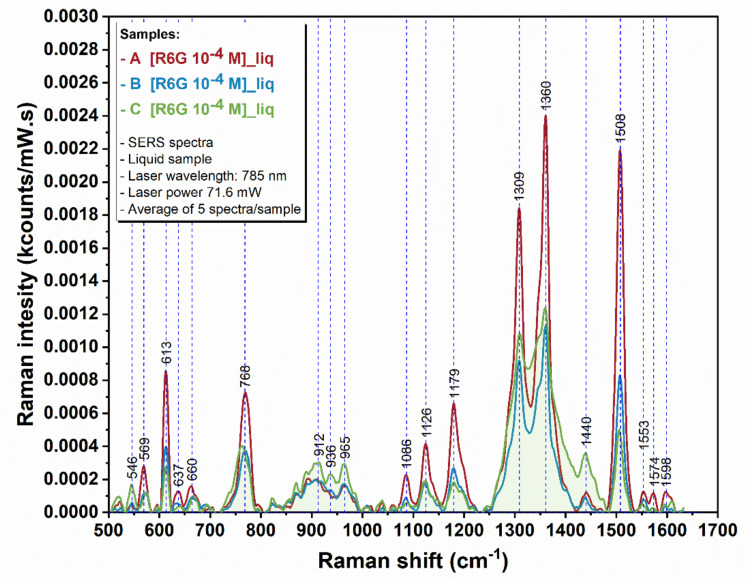
SERS spectra of R6G in colloidal dispersion of GNBs (samples A and B) and GNRs (sample C).

SERS performances of on-resonance gold nanostructures with different morphologies and similar aspect ratios were compared in colloidal dispersion (Figure 7). It was found that GNBs (sample B) have superior activity compared to GNRs (sample C).

**Figure 8:**
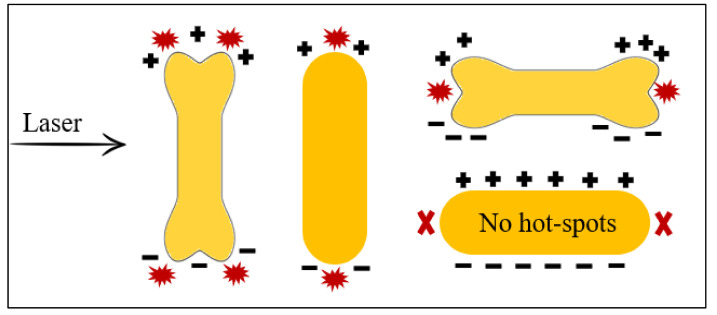
Schematic diagram of surface charge distribution in GNBs and GNRs, oriented parallel or perpendicular to the electric field. Adapted from [13].

This observation might be explained by the interplay of several factors. The main cause of Raman signal augmentation is the high curvature of the GNR’s ends (lightning rod effect). This implies that, for a maximum enhancement of the electromagnetic field surrounding the particle (Figure 8), the excitation laser’s direction must be perpendicular to the long axis of the GNRs. In dispersion, due to the random orientation of the GNRs, the enhancement of the signal is relatively low. In contrast to GNRs, GNBs amplify the signal regardless of laser’s orientation [13]. When the laser is parallel to the long axis of the GNBs, hot-spots are formed between the lobes at the ends with the same favorable effect on the recorded SERS signal. The sharper edges of GNBs in comparison with GNRs might be another factor responsible for the superior SERS activity of GNBs in dispersion [36]. Finally, the size of the nanostructures has also a substantial influence on signal enhancement, as it was demonstrated that smaller nanorods generate stronger local electromagnetic fields [37] and thus, higher SERS signals, due to a stronger lightning rod effect and a weaker radiation damping.

SERS performances of blue-shifted, off-resonance GNBs (sample A) were compared to on-resonance nanostructures in dispersion. As a result, an even stronger enhancement of the Raman signal was observed for sample A compared to both samples B and C (~2- and 6-fold increase, respectively; Table S1). In this case, the better performances of GNB nanostructures can be assigned to the competition at the resonance frequency between different optical phenomena.

**Figure 9:**
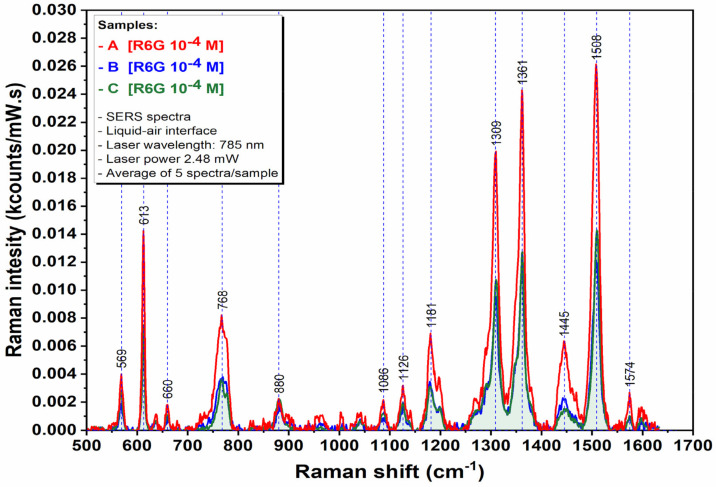
SERS spectra of R6G trapped at liquid-air interface in GNBs (samples A and B) and GNRs (sample C) films.

Studies at the liquid interface showed that the Raman signal can be further enhanced by the aggregation of nanostructures in films. By recording the SERS signal of R6G 0.1 mM at the liquid-air interfacial region, a 17, 18, and 55-fold increase (Table S1) of Raman intensity is observed for samples A, B, and C, respectively (Figure 9) as compared to the colloidal systems.

If in dispersion the signal for on-resonance GNBs is three times higher than for on-resonance GNRs, at the liquid interface, the shape particularities of the gold nanostructures are no longer critical, the recorded signal being more similar. The remarkable signal increase (55-fold) using GNRs at the liquid interface vs. in dispersion might be explained by the resulting array of hot-spots between the nanostructures upon different types of interactions: end-to-end, side-by-side, or tip-to-side.

Because the recorded SERS signal was far superior for blue-shifted GNRs (sample A vs. B and C) both in dispersion (2.13 and 6.19-fold) and at interface (2.03 and 1.88-fold) (Table 2), it can be concluded that tuning nanostructures’ plasmonic peak (aspect ratio) is more important than nanostructure’s shape, especially in the case of interfacial region. This is somewhat in contrast to the findings of other authors measuring the SERS response in colloidal GNRs and dumbbells, where tuning the NPs shape was considered more important [38].

A good reproducibility of the recorded R6G Raman signal intensity (%RSD, Table 2) has been obtained, regardless of the sampled area of the film from the interfacial region. Moreover, for the developed blue-shifted GNBs film at the interface, a 5 nM limit of detection for R6G (Figure S3) has been measured.

## 4. Conclusions

Comparing the SERS enhancement efficiency of different nanostructures, we found that off-resonance GNBs are superior to on-resonance GNBs and GNRs in both colloidal dispersion and aggregated close-packed films formed at the liquid-liquid interface. More research is needed in order to solve the inconsistency regarding the link between the position of the plasmonic peaks and Raman enhancement. By aggregating nanostructures at the liquid interface, we recorded a 16-, 17-, and 55-fold SERS signal enhancement, regardless of the region of the film where the measurements were performed. The Raman signal intensity was very reproducible, and excellent standard deviation values are reported. One can also conclude that upon aggregation at the liquid interface, the shape of nanostructures is not as important as tuning the plasmonic peaks. When off-resonance GNBs were assembled at the liquid interface, a relative standard deviation of 4.56% and a limit of detection of 5 nM was obtained for R6G, rendering this substrate suitable for analytical applications. Building nanoplatforms at the liquid interface is advantageous (better stability compared to suspended aggregates, reduced time for detection, and a simplified process, as the film developed at the liquid interface does not have to be transferred on to a solid substrate) and leads to reliable nanoplatforms for sensing applications.

## Figures and Tables

**Table 1:** Optical properties of the studied anisotropic gold nanostructures.

Sample	A	B	C
Longitudinal SPR peak (nm)	710	765	765
Middle SPR peak (nm)	582	602	-

**Table 2:** Raman intensity and standard deviation for samples A, B, and C in dispersion and as aggregated films at the liquid-air interfacial region.

Media	Sample	Raman Intensity at 612 cm^−1^ (kcounts/mW·s)	RSD%
**Colloidal dispersion**	A	0.000858	±2.43%
	B	0.000401	±3.03%
	C	0.000139	±0.87%
**Liquid interface**	A	0.014408	±4.56%
	B	0.007078	±13.59%
	C	0.007661	±8.44%

## Data Availability

The data that support the findings of this study are available from the corresponding author upon reasonable request.

## Supplementary Materials

The following are available online at https://www.mdpi.com/article/10.3390/s22010236/s1, Figure S1: Chemical structure (left) and corresponding Raman spectrum of R6G (right), Figure S2: Images of the close-packed films formed via self-assembly at the CYH/Water interface seen through optical microscope, Figure S3: SERS spectra of 5 nM R6G on off-resonance GNBs film assembled at liquid-liquid interface. Characteristic vibrational bands can still be distinguished at 612 cm^−1^ (C–C–C ring in-plane vibration), 1311 cm^−1^ (N–H in-plane bending), 1362 cm^−1^ and 1507 cm^−1^ (C–C stretching), Table S1: Enhancement ratio for samples A, B and C in dispersion (“d”) and as aggregated films at the liquid-air interfacial region (“i”).
