# Supplementary material for: Off-Resonance Gold Nanobone Films at Liquid Interface for SERS Applications
Source: Sensors (Basel). 2021 Dec 29;22(1):236. doi: 10.3390/s22010236 (PMC8749543; doi:10.3390/s22010236)
Supplement: Supplementary file 1 [file sensors-22-00236-s001.zip › sensors-1514656-supplementary.pdf]

Supplementary

Off-Resonance Gold Nanobone Films at Liquid Interface for SERS Applications

Rebeca Moldovan [1], Valentin Toma [2], Bogdan-Cezar Iacob [1], Rareș Ionuț Știufiuc [2,3] and Ede Bodoki [1,*]

[1] Analytical Chemistry Department, Faculty of Pharmacy, Iuliu Hațieganu University of Medicine and Pharmacy, 400349 Cluj-Napoca, Romania; rebeca.magda@umfcluj.ro (R.M.); iacob.cezar@umfcluj.ro (B.-C.I.)

[2] MedFuture Research Center for Advanced Medicine, "Iuliu Hațieganu" University of Medicine and Pharmacy,400349 Cluj-Napoca, Romania; valentin.toma@umfcluj.ro (V.T.); rares.stiufiuc@umfcluj.ro (R.I.Ș.)

[3] Pharmaceutical-Biophysics Department, Faculty of Pharmacy, Iuliu Hațieganu University of Medicine and Pharmacy, 400349 Cluj-Napoca, Romania

* Correspondence: bodokie@umfcluj.ro

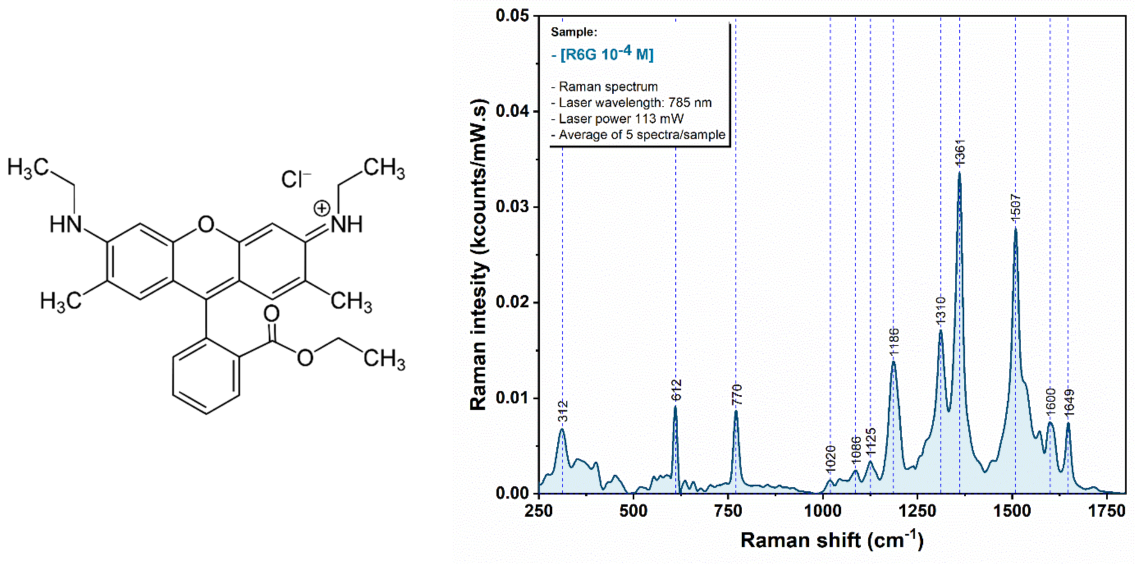

Figure S1. Chemical structure (left) and corresponding Raman spectrum of R6G (right).

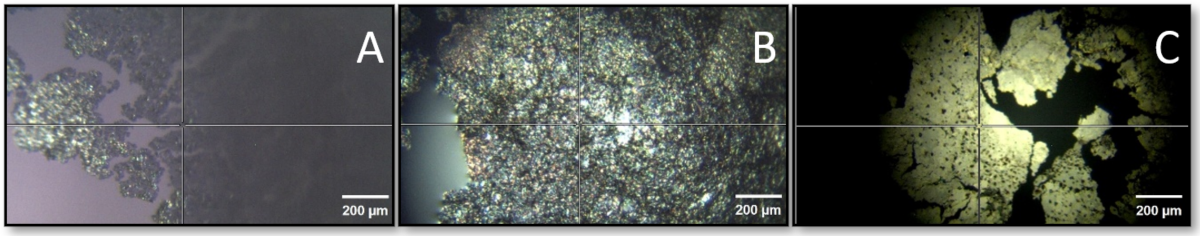

Figure S2. Images of the close-packed films formed via self-assembly at the CYH/Water interface seen through optical microscope.

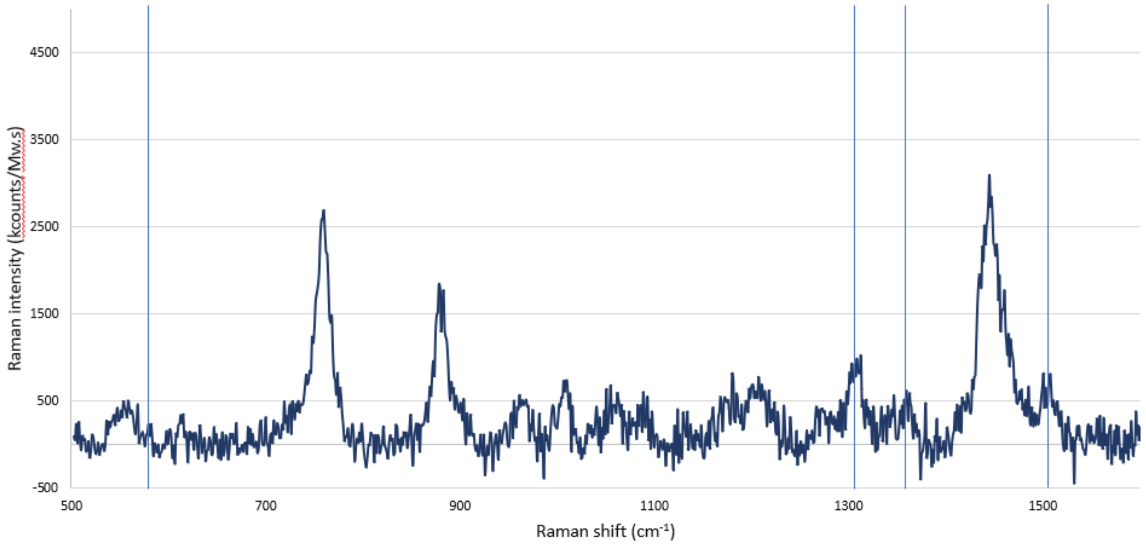

Figure S3. SERS spectra of 5 nM R6G on off-resonance GNBs film assembled at liquid-liquid interface. Characteristic vibrational bands can still be distinguished at 612 cm^{-1} (C–C–C ring in-plane vibration), 1311 cm^{-1} (N–H in-plane bending), 1362 cm^{-1} and 1507 cm^{-1} (C–C stretching).

Table SI. Enhancement ratio for samples A, B and C in dispersion (“d”) and as aggregated films at the liquid-air interfacial region (“i").

**Raman Intensity Ratio**	**Raman Shift 612 cm^{-1}**
Ad/Bd	2.13
Ad/Cd	6.19
Bd/Cd	2.89
Ai/Bi	2.03
Ai/Ci	1.88
Bi/Ci	0.92
Ai/Ad	16.79
Bi/Bd	17.65
Ci/Cd	55.31
